# A Somatic Mutation-Derived LncRNA Signature of Genomic Instability Predicts Prognosis for Patients With Liver Cancer

**DOI:** 10.3389/fsurg.2021.724792

**Published:** 2021-08-24

**Authors:** Cheng Guo, Jie Zhou, Boyu Ma, Rui Wang, Yanli Ge, Zhe Wang, Bing Ji, Wei Wang, Junjie Zhang, Zhirong Wang

**Affiliations:** Department of Gastroenterology, Tongji Hospital, Tongji University School of Medicine, Shanghai, China

**Keywords:** hepatocellular carcinoma, long non-coding RNA, immune infiltration, prognosis, genomic instability

## Abstract

**Background:** Genomic instability is considered as one of the hallmarks of hepatocellular carcinoma (HCC) and poses a significant challenge to the clinical treatment. The emerging evidence has revealed the roles of long non-coding RNAs (lncRNAs) in the maintenance of genomic instability. This study is aimed to develop a genomic instability-related lncRNA signature for determining HCC prognosis and the suitability of patients for immunotherapy.

**Methods:** In this study, data related to transcriptome profiling, clinical features, and the somatic mutations of patients with HCC were downloaded from The Cancer Genomic Atlas (TCGA). Bioinformatics analysis was performed to identify and construct a somatic mutation-derived genomic instability-associated lncRNA signature (GILncSig). Single-sample gene set enrichment analysis (ssGSEA) was applied to estimate the levels of immune cell infiltration. A nomogram was constructed, and calibration was performed to assess the effectiveness of the model.

**Results:** In the study, seven genomic instability-related lncRNAs were identified and used to define a prognostic signature. Patients with HCC were stratified into high- and low-risk groups with significant differences in the survival (median survival time = 1.489, 1.748 year; *p* = 0.006) based on the optimal cutoff value (risk score = 1.010) of the risk score in the training group. In addition, GILncSig was demonstrated to be an independent risk factor for the patients with HCC when compared to the clinical parameters (*p* < 0.001). According to the receiver operating characteristic (ROC) curve, nomogram, and calibration plot, the signature could predict the survival rate for the patients with HCC in the 1st, 3rd, and 5th years. Furthermore, ssGSEA revealed the potential of the signature in guiding decisions for administering clinical treatment.

**Conclusions:** In this study, we developed a novel prognostic model based on the somatic mutation-derived lncRNAs and validated it using an internal dataset. The independence of the GILncSig was estimated using univariate and follow-up multivariate analyses. Immunologic analysis was used to evaluate the complex factors involved in the HCC progression.

## Background

Hepatocellular carcinoma (HCC) is a malignant tumor with a high rate of recurrence and poor prognosis, constituting the fourth major cause of cancer-associated deaths worldwide (1). Surgical resection, transplantation, and radiofrequency ablation are the most effective treatment methods for patients with early-stage liver cancer (1). For a decade, sorafenib, an antiangiogenic tyrosine kinase inhibitor, is the only treatment strategy recognized by the Food and Drug Administration (FDA) for advanced liver cancer (2). However, the therapeutic efficacy of sorafenib is gradually weakening or restricting owing to the chemical resistance and recurrence (3, 4). Early diagnosis is essential for improving patient outcomes (4). The Liver Cancer Staging System, described in the eighth edition of the American Joint Committee on Cancer (AJCC 2017), is one of the most recognized staging systems globally (5). However, the accuracy of AJCC staging in predicting prognosis in patients with liver cancer requires improvement.

Genomic instability attributed to the somatic mutations is a hallmark of cancer cells (6). Genomic instability is observed frequently in HCC (6, 7). It is also a significant prognostic parameter, and an increase in the genomic instability ratio indicates a worse outcome (8–10). Although the mechanisms underlying genomic instability are not entirely clear, aberrant transcription and post-transcriptional modifications play important roles (11).

Non-coding RNAs with a length of more than 200 nucleotides, termed long non-coding RNAs (lncRNAs), are characterized as non-coding transcripts and do not encode proteins (12). The abnormal regulation, involving processes such as deletion or mutation, of lncRNA has been associated with many human diseases, including cancer (13). More importantly, lncRNAs are involved in chromatin interactions, transcriptional regulation, mRNA post-transcriptional regulation, and epigenetic regulation. New evidence has illustrated the vital roles of lncRNAs in regulating genomic stability (14–17). For example, Lee et al. (17) identified a specific non-coding RNA, NORAD, which alters genomic stability through the sequestration of PUMILIO proteins. Although several lncRNAs are associated with genomic instability, the regulatory role of lncRNAs associated with genomic instability in cancers remains elusive.

In this study, we developed a novel promising prognostic signature that is more effective than the AJCC staging system. In addition, our research showed the immune microenvironment and immune functions of patients with HCC.

## Methods

### Data Source

RNA-seq expression data (FPKM), somatic mutation information, and the related clinical variables of 343 patients with liver cancer were obtained from The Cancer Genomic Atlas (TCGA, http://cancergenome.nih.gov/, accessed July 20, 2020). The lncRNA expression profile was extracted from the mRNA expression profile data based on the GTF file information downloaded from the GENECODE website.

### Identification and Construction of the Genomic Instability-Associated lncRNA Signatures

**Figure 1:**
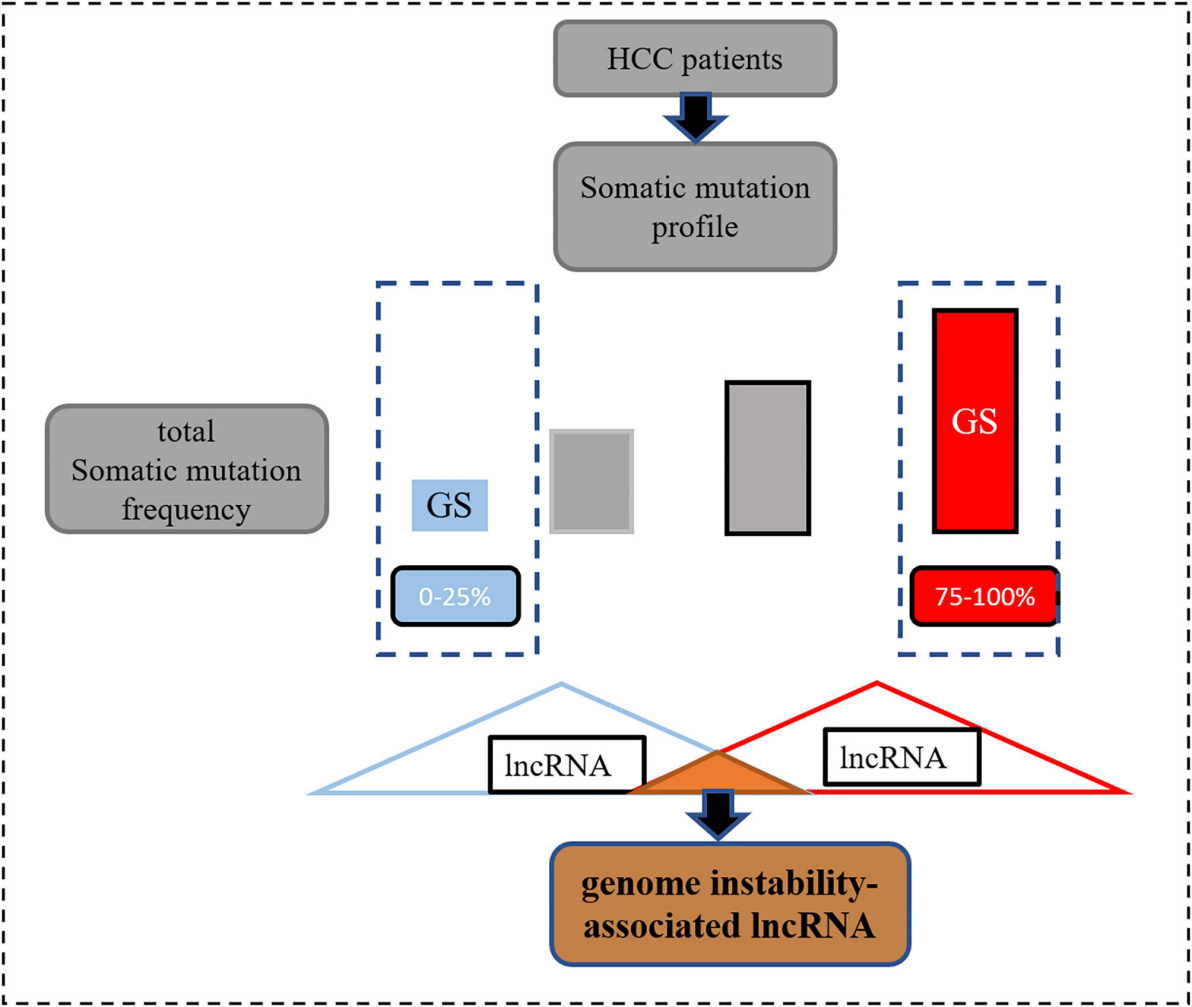
The process of screening genomic instability-associated lncRNAs.

Information on somatic variants observed in patients with HCC stored in the format of mutation annotation downloaded from TCGA was analyzed using the “maftools” R package (17). In order to identify the genomic instability-associated lncRNAs, first, after calculating the total number of mutations per patient, they were ranked in descending order (Figure 1). Second, according to a previous study (18), the top 25% and the last 25% were characterized as the genomic unstable (GU) group and genomic stable (GS) group, respectively. Third, the Wilcoxon test was employed for the comparison of the lncRNA expression matrix between the GU and GS groups. Finally, according to the definition, differentially expressed lncRNAs [log_2_ fold change >*|*1| and false discovery rate (FDR) adjusted *p* < 0.05] were considered as the genomic instability-related lncRNAs.

Among the genomic instability-related lncRNAs, lncRNAs associated with the overall survival (OS) of patients with HCC were screened by employing univariate Cox regression analyses (*p* < 0.05). Next, the genomic instability-associated lncRNA signature (GILncSig) was constructed by performing multivariate Cox regression analysis. The risk score was determined for every patient by applying the following formula: GILncSig score = coef_lncRNA1_^*^ expr_lncRNA1_ + coef_lncRNA2_^*^ expr_lncRNA2_ + … + coef_lncRNAn_^*^ expr_lncRNA*n*_, based on a previous study (18). Linear integration of the expression levels of lncRNAs weighted by regression coefficients (coef) was used to assign the risk score. Log transformation of the hazard ratio (HR) from the multivariate Cox regression analysis was employed to calculate the coef value. Patients enrolled in the training group were divided into high and low-risk groups using the median score as a cutoff value. The Kaplan-Meier method was used to compare the survival rates between the high-risk and low-risk groups. The independence of the risk signature was estimated from other clinical features using the multivariate Cox regression.

### ssGSEA Analysis

The immune-related term enrichment score was established by exploring the associations between the risk score of GILncSig and components of the immune system, such as innate or adaptive immune cell types, immune functions, or pathways using the ssGSEA program. The levels of immune infiltration were estimated using the “gsva” R package (19). Quantitative indicators of immune infiltration were determined for each patient with HCC. In addition, different distributions of immune cell types or functions between the high and low-risk groups in the TCGA cohort were visualized using the “vioplot” R package (20).

### Construction and Assessment of a Predictive Nomogram

A nomogram was constructed based on the clinical factors using multivariate regression analysis in the training group. The discrimination and calibration of the predictive nomogram were assessed by applying the concordance index (C-index) and the calibration curve. The construction of nomogram and calibration was performed using the “rms” package (21). The effectiveness of the signature was assessed by constructing a time-dependent receiver operating feature (ROC) curve using the “survivalROC” packages (22).

### Statistical Analysis

All statistical analyses were performed using the RStudio (v.1.4.1106). Continuous data are presented as medians or mean ± SD. Statistical significance was set at *p* < 0.05.

## Results

### Identifying the Genomic Instability-Related lncRNAs in Patients With HCC

**Figure 2:**
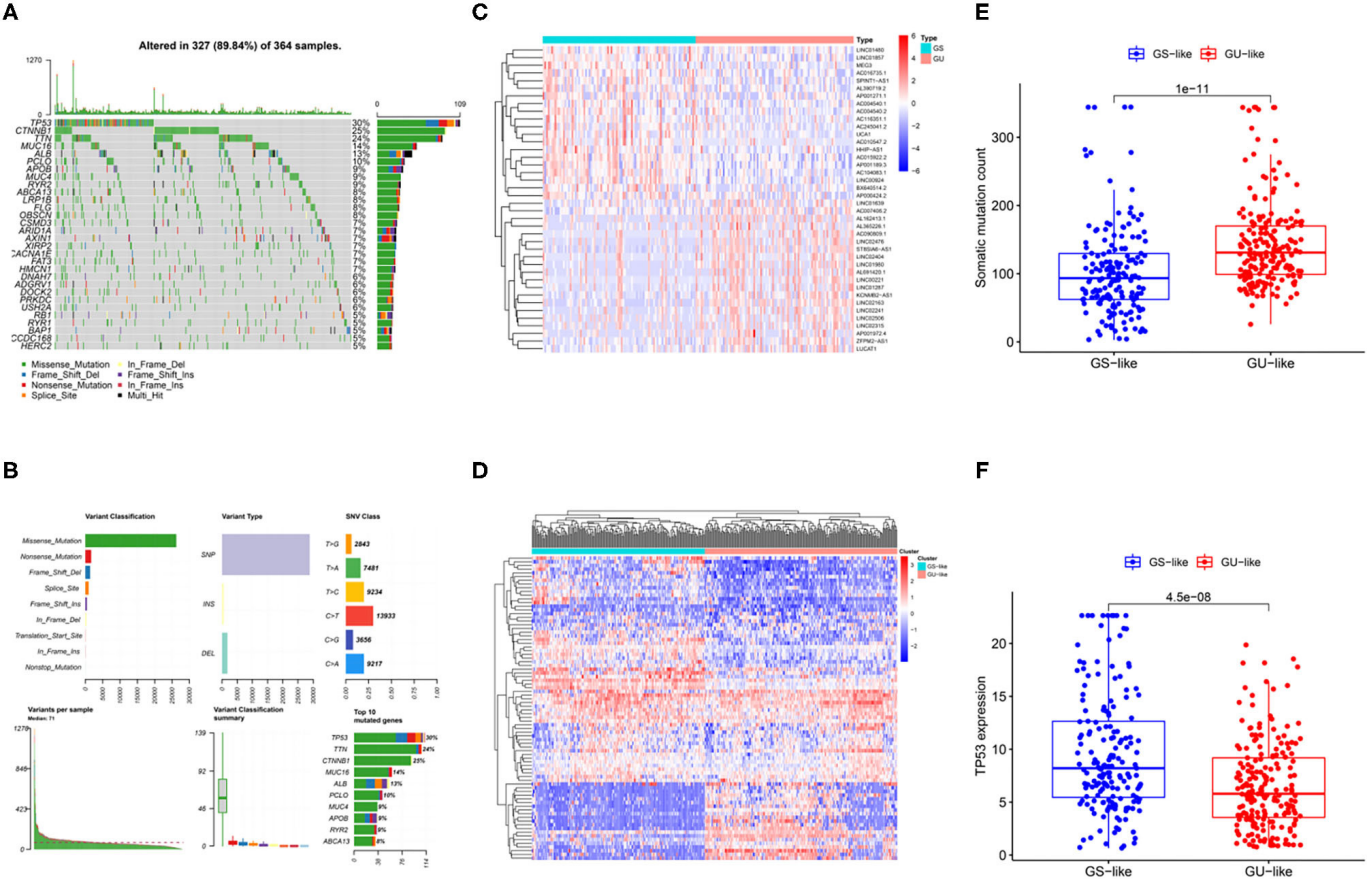
Identification of genomic instability-related lncRNAs in patients with HCC. **(A)** Waterfall summarized mutational data from patients with HCC. **(B)** Mutational frequencies in the top 10 genes in the training and testing cohorts. **(C)** Differentially expressed lncRNAs between GS group and GU group. **(D)** Hierarchical cluster analyses of TCGA patients with liver cancer based on the expression pattern of 82 genomic instability-related lncRNAs. **(E)** Boxplots of somatic mutations in the GU-like and GS-like groups. **(F)** Boxplots of TP53 expression level in the GU-like and GS-like groups.

Liver cancer is highly heterogeneous with respect to the mutated genes (23, 24). In Figures 2A,B, we illustrate the landscape of the HCC mutation profile including variant classification, type of variants, single nucleotide polymorphism (SNP) class, variants per sample, and the top 10 mutated genes. As shown in Figure 2B, SNP was the most common variant type. TP53 was the most mutated gene in the HCC, with an average of 30% mutation frequency. To identify lncRNAs related to the genomic instability, differential lncRNA expression profiles of patients with HCC between the GU and GS groups determined by different mutation patterns were compared. Wilcoxon's test was used to screen 82 lncRNAs with significant differences (log_2_ fold change > |1| and FDR regulated *p* < 0.05; Supplementary Table 1). The expression of 53 of 82 lncRNAs was upregulated and that of the remaining lncRNAs was downregulated. The profiles of the top 20 lncRNAs with upregulated and downregulated expressions are shown in Figure 2C. In addition, all the patients were divided into two clusters based on the differential expression profiles of the 82 lncRNAs (Figure 2D). The group with higher somatic mutation frequency was regarded as the GS-like group, while the other group was regarded as the GU-like group. The somatic mutation patterns of the two groups were significantly different. Cumulative somatic mutations in the GS-like group were significantly lower than those in the GU-like group (Figure 2E, *p* < 0.001). In addition, the TP53 expression was significantly higher in the GS-like group than that in the GU-like group (Figure 2F, *p* < 0.001).

### Construction of the Genomic Instability-Related lncRNA Signature

To investigate the prognostic effectiveness of these genomic instability-related lncRNAs, 343 patients with liver cancer from the TCGA database were randomly assigned to the training group (*n* = 172) and the testing group (*n* = 171). The inclusion standard included patients with complete prognostic information and pathologically confirmed HCC. The exclusion criterion was patients with equal to or <30 days of survival because they were more likely to die from non-tumor factors, such as post-operative bleeding or infection. No statistically significant differences were observed with respect to age (*p* = 0.855), gender (*p* = 0.538), AJCC stage (*p* = 0.170), histologic grade (*p* = 1), T (*p* = 0.239), M (*p* = 1), and N (*p* = 1) using the chi-square test (Table 1).

**Table 1:** Clinical information of the 343 patients with liver cancer.

**Variables**	**Type**	**Entire group (***n*** = 343)**	**Testing group (***n*** = 172)**	**Training group (***n*** = 171)**	***p*** **-value**
Age	< =65	216 (62.97%)	109 (63.74%)	107 (62.21%)	0.8554
	>65	127 (37.03%)	62 (36.26%)	65 (37.79%)	
Gender	Female	110 (32.07%)	58 (33.92%)	52 (30.23%)	0.5382
	Male	233 (67.93%)	113 (66.08%)	120 (69.77%)	
Histologic grade	G1-2	214 (62.39%)	106 (61.99%)	108 (62.79%)	1
	G3-4	124 (36.15%)	62 (36.26%)	62 (36.05%)	
	Unknown	5 (1.46%)	3 (1.75%)	2 (1.16%)	
AJCC stage	Stage I–II	238 (69.39%)	112 (65.5%)	126 (73.26%)	0.1695
	Stage III–IV	83 (24.2%)	47 (27.49%)	36 (20.93%)	
	Unknown	22 (6.41%)	12 (7.02%)	10 (5.81%)	
T	T1-2	252 (73.47%)	120 (70.18%)	132 (76.74%)	0.2386
	T3-4	88 (25.66%)	49 (28.65%)	39 (22.67%)	
	Unknown	3 (0.87%)	2 (1.17%)	1 (0.58%)	
M	M0	245 (71.43%)	121 (70.76%)	124 (72.09%)	1
	M1	3 (0.87%)	1 (0.58%)	2 (1.16%)	
	Unknown	95 (27.7%)	49 (28.65%)	46 (26.74%)	
N	N0	239 (69.68%)	118 (69.01%)	121 (70.35%)	1
	N1	2 (0.58%)	1 (0.58%)	1 (0.58%)	
	Unknown	102 (29.74%)	52 (30.41%)	50 (29.07%)	

**Figure 3:**
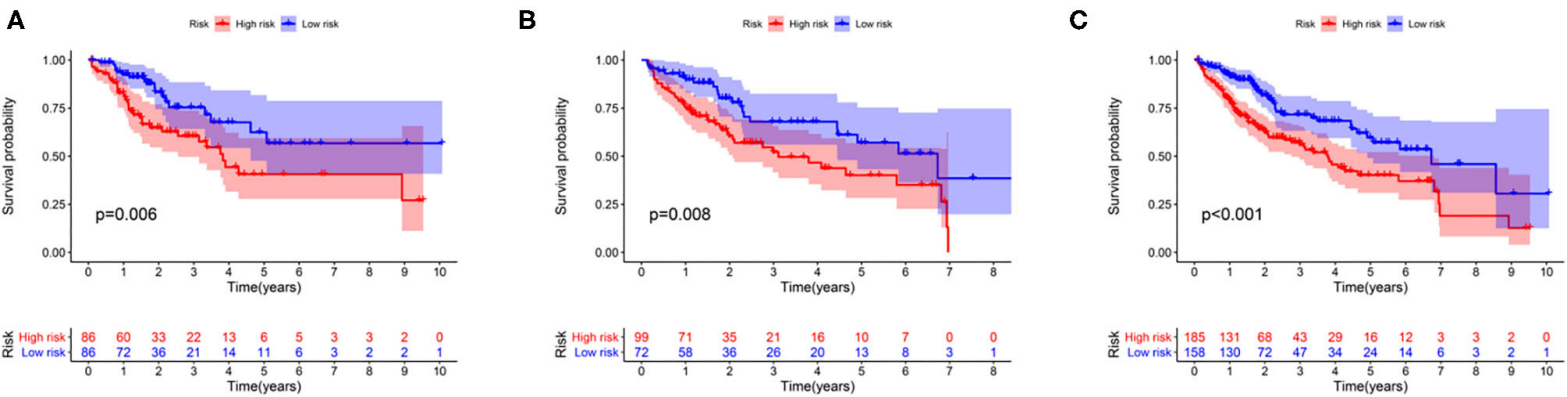
Survival curve analysis between the high-risk and low-risk groups in the training group **(A)**, the testing group **(B)**, and the entire group **(C)**.

The association between the expression profiles of 82 genomic instability-related lncRNAs and the OS of patients with liver cancer in the training group was analyzed using the univariate Cox proportional hazard regression. The results revealed that 11 lncRNAs influenced the prognosis of patients with HCC (*p* < 0.05; Supplementary Table 2). These lncRNAs were then incorporated into a multivariate Cox proportional hazards regression model to identify an optimal risk signature model without the risk of overfitting using the “glmnet” R package. Finally, 7 of 11 lncRNAs were used to construct the GILncSig based on the maximum value of the Akaike information criterion (AIC, AIC = 446.94) (Table 2). The GILncSig score was calculated using the following formula: value of LINC01287^*^0.035+ value of AC004540.1^*^0.259+ value of AC096996.2^*^0.338+ value of PRRT3-AS1^*^0.202+ value of AC004862.1^*^(−0.188) + value of AC245041.2^*^0.063+ value of AC010205.1^*^(−0.782). The coefficients of these lncRNAs represent the contribution of lncRNAs to the prognostic risk score obtained from the regression index of multivariate Cox analysis. The aforementioned formula was adopted to obtain the risk score of patients with liver cancer in the training group, and the median risk score (value = 1.010) was used as the cutoff value to cluster these patients into different groups. The group with a higher score was called the high-risk group, and the other group was called the low-risk group. The Kaplan-Meier analysis indicated that patients in the low-risk group showed better outcomes than those in the high-risk group (*p* = 0.006; *p* = 0.008; *p* < 0.001) (Figure 3).

**Table 2:** Seven lncRNAs identified using the Cox regression model.

**LncRNA**	**Coefficient**	**Hazard ratio (95% CI)**	***p-*** **value**
LINC01287	0.035	1.036 (1.002–1.071)	0.039
AC004540.1	0.259	1.296 (1.029–1.632)	0.028
AC096996.2	0.338	1.403 (1.065–1.847)	0.016
PRRT3-AS1	0.202	1.224 (1.091–1.374)	0.001
AC004862.1	−0.188	0.828 (0.663–1.035)	0.098
AC245041.2	0.063	1.066 (0.993–1.414)	0.076
AC010205.1	−0.782	0.457 (0.206–1.018)	0.055

**Figure 4:**
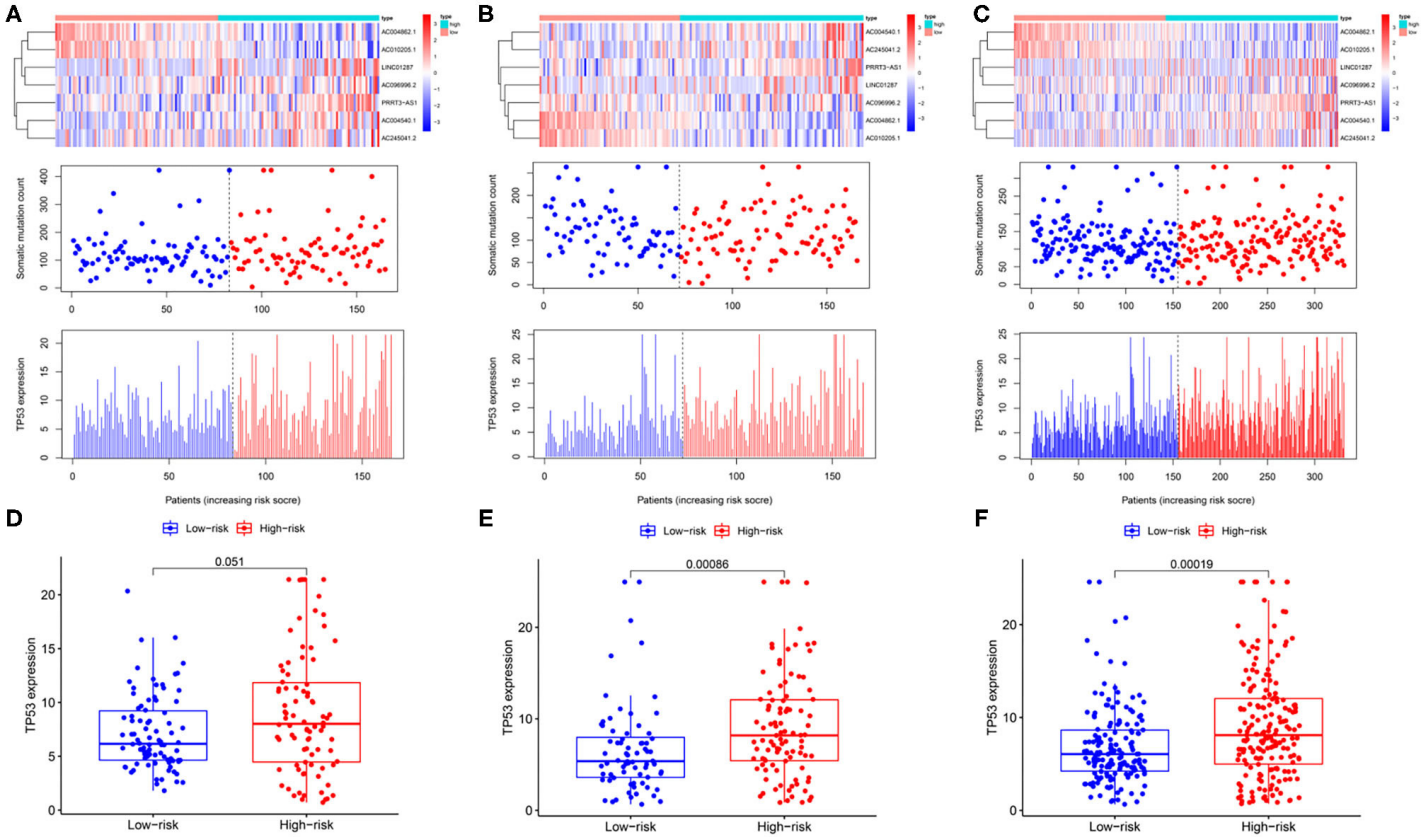
Identification of the genomic instability-related lncRNA signature for outcome prediction. Heatmap of LncRNAs and the distribution of somatic mutation and TP53 expression with increasing risk score in the training group **(A,D)**, in the testing group **(B,E)**, and the entire group **(C,F)**.

The expression profiles of lncRNAs, the distribution of somatic mutation frequency, and TP53 expression in different cohorts are illustrated in Figures 4A–C. TP53 expression was significantly higher in the high-risk group than that in the low-risk group in the testing cohort as well as the entire cohort (*p* < 0.001; Figures 4E,F). Although the level of TP53 was not significantly different between the high-and low-risk groups in the training cohort, the *p-*value was close to 0.05 (Figure 4D).

### Independence of the GILncSig From Other Clinical Parameters

**Figure 5:**
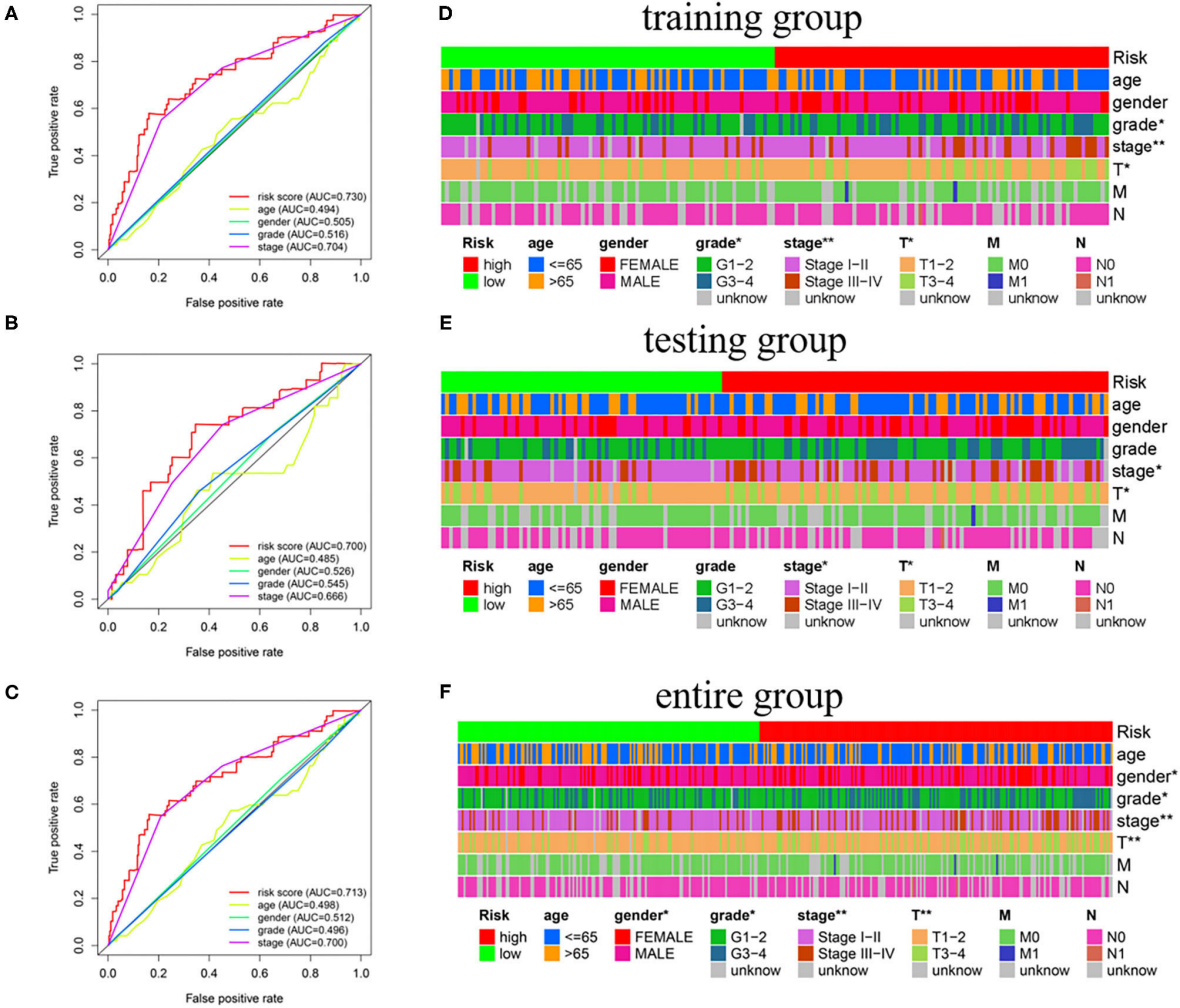
The association of GILncSig and clinical features. **(A**–**C)** ROC curve analysis of the training group, the testing group, and the entire group. **(D**–**F)** Differential analysis between GILncSig and clinicopathological features. ^*^p < 0.05; ^**^p < 0.01.

The independence of the GILncSig in patients with HCC was assessed by performing Cox regression analyses. The univariate and follow-up multivariate analyses showed that AJCC stage (*p* = 0.002; *p* = 0.002; *p* < 0.001) and GILncSig risk scores (*p* < 0.001; *p* < 0.001; *p* = 0.002) were significantly related to OS and were independent factors for patients with liver cancer in the three groups (Table 3). In addition, according to the time-dependent ROC curve analysis, the area under the curve (AUC) for the risk score was 0.730, which was higher than the AUC values for age (AUC = 0.494), gender (AUC = 0.505), grade (AUC = 0.516), and stage (AUC = 0.704) in the training group (Figure 5A). The testing set and the entire set showed similar outcomes (Figures 5B,C).

**Table 3:** The univariate and multivariate Cox regression analyses in the training, testing, and entire groups.

**Variable**	**Univariate analysis**			**Multivariate analysis**		
	**HR**	**HR.95L-HR.95H**	***p***	**HR**	**HR.95L-HR.95H**	***p***
**Training set** (***n*** **= 172)**						
Age (<=65/>65)	1.005	0.983–1.027				
Gender (Female/male)	0.870	0.480–1.578				
Grade (G1–G4)	0.802	0.535–1.204				
Stage (I–IV)	1.776	1.293–2.440	**<0.001**	1.665	1.205–2.300	**0.002**
Risk score	1.131	1.087–1.177	**<0.001**	1.123	1.077–1.170	**<0.001**
**Testing set** (***n*** **= 171**)						
Age (<=65/>65)	1.006	0.986–1.026				
Gender (Female/male)	0.696	0.413–1.174				
Grade (G1–G4)	1.460	1.037–2.056				
Stage (I–IV)	1.783	1.339–2.375	**0.030**	1.609	1.160–2.205	**0.002**
Risk score	1.009	0.980–1.038	**<0.001**	1.229	1.172–1.280	**<0.001**
**Entire set** (***n*** **= 343**)						
Age (<=65/>65)	1.005	0.991–1.020				
Gender (Female/male)	0.758	0.513–1.118				
Grade (G1–G4)	1.121	0.865–1.454				
Stage (I–IV)	1.808	1.463–2.234	**<0.001**	1.775	1.436–2.194	**<0.001**
Risk score	1.028	1.010–1.047	**0.003**	1.025	1.005–1.046	**0.002**

*HR, hazard ratio. Bold value means p< 0.05*.

Moreover, we investigated the association between the independent signature and clinical parameters by using the chi-square test. Tumor grade (*p* < 0.05), AJCC stage (*p* < 0.01), and T stage (*p* < 0.05) were significantly related to GILncSig in the training group (Figure 5D). Notably, the AJCC stage and the T stage were significantly associated with the GILncSig in the training, testing, and overall groups (Figures 5D–F).

### Association Between the GILncSig and Immune Cell Infiltration

**Figure 6:**
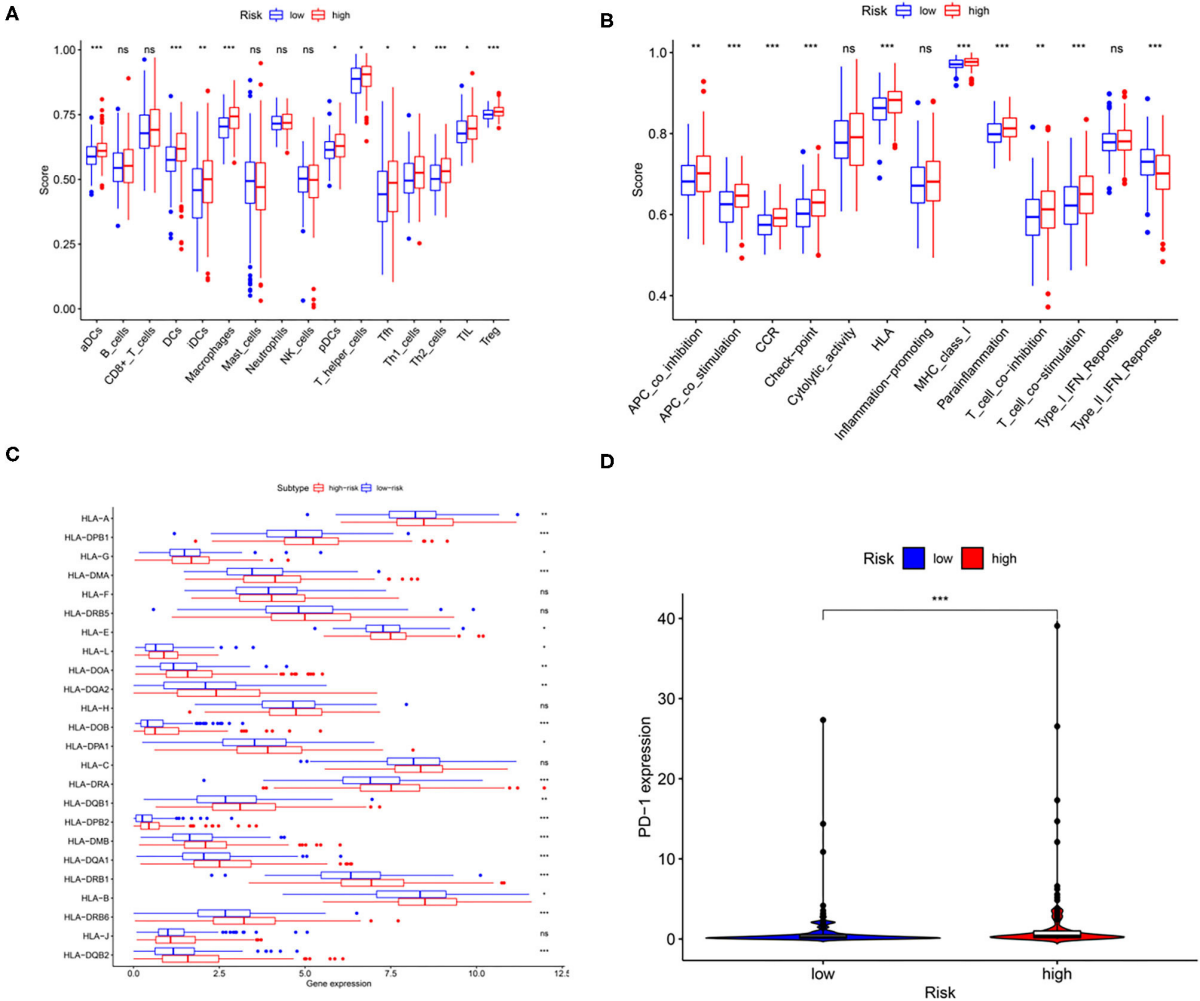
The relationship among the risk scores, immune cell infiltration, and immune functionality in patients with liver cancer. The relative enrichment of immune cell infiltration **(A)** and immune functionality **(B)** in the patients with high- and low-risk liver cancer. HLA family gene **(C)** and PD-1 expression **(D)** in patients with high- and low-risk liver cancer. ^*^p < 0.05; ^**^p < 0.01; ^***^p < 0.001.

In the study, immune cell types, immune functions, or pathways were evaluated to assess immune cell infiltration among patients with HCC in an integrated fashion *via* ssGSEA analysis of the transcriptome profiles of patients with liver cancer. As shown in Figures 6A,B, GILncSig scores showed a significantly positive association with immune cell types, including various types of dendritic cells, T-helper cells, Treg cells, follicular helper T cells, macrophages, and tumor-infiltrating lymphocytes. In addition, patients in the high-risk group showed a higher proportion of APC co-stimulation, APC co-inhibition, CCR, check-point, HLA, MHC class I, para inflammation, T-cell co-stimulation, and T-cell co-inhibition. In addition, seven of the 13 types of immune functions were significantly higher in high-risk patients than in low-risk patients, while the expression of type II IFN response was the opposite. More importantly, the expression of HLA family genes and programmed cell death protein-1 (PD-1) in the high-risk group was significantly higher than that in low-risk patients with HCC (Figures 6C,D). The aforementioned results showed that abnormal immune infiltration and differences in the expression of immune checkpoints can be adopted as prognostic indices for patients with liver cancer with respect to immunotherapy with significant clinical implications.

### Nomogram Construction and Validation

**Figure 7:**
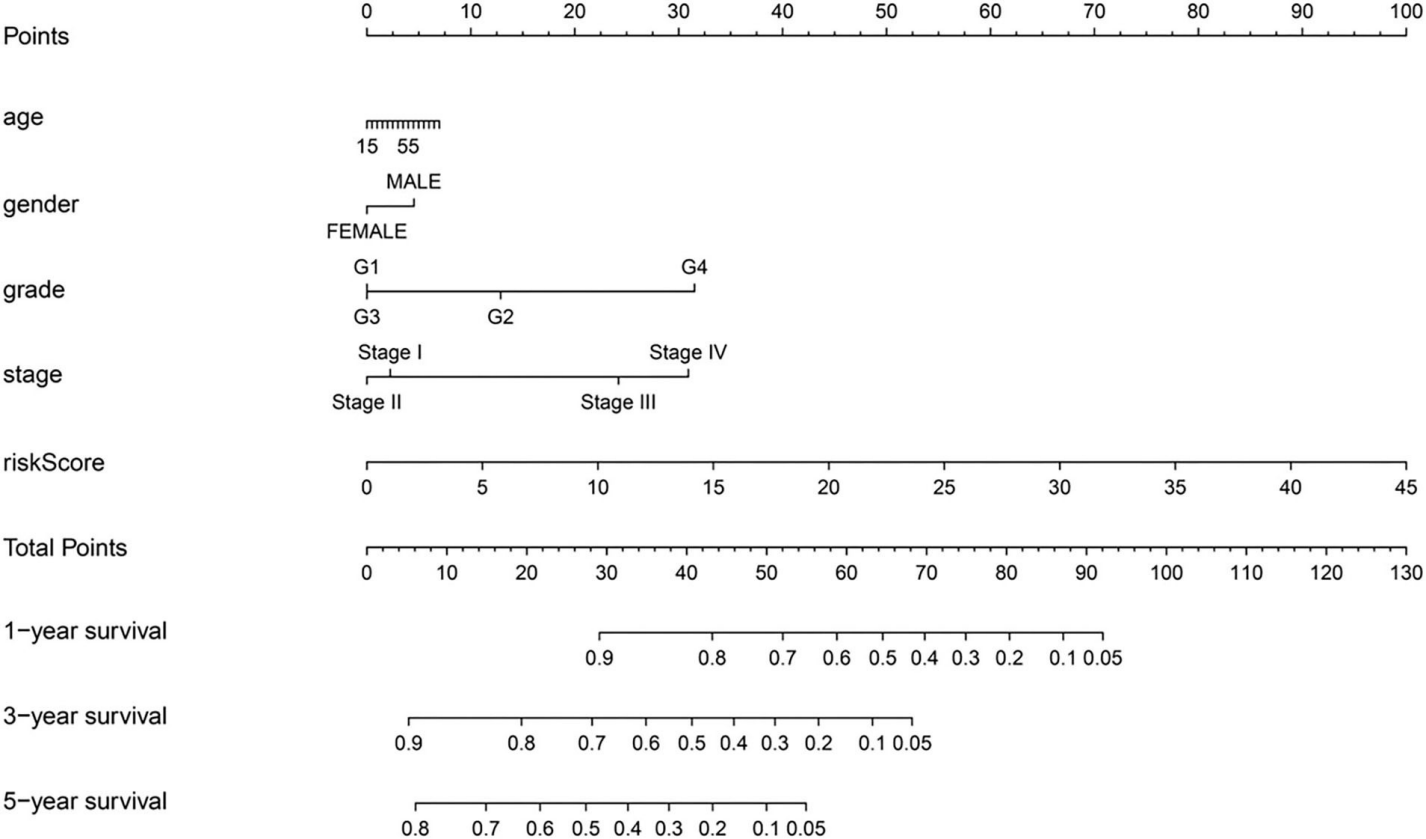
A predictive nomogram for OS in the training group.

A nomogram was constructed by combining the age, gender, tumor grade, AJCC stage, and risk score (Figure 7). Each parameter in the nomogram was assigned a score. Based on the parameters of each patient, the score related to each prognostic element was added to obtain the total score, which corresponds to the corresponding scale. The survival rates of the patients were obtained at the 1st, 3rd, and 5th years.

**Figure 8:**
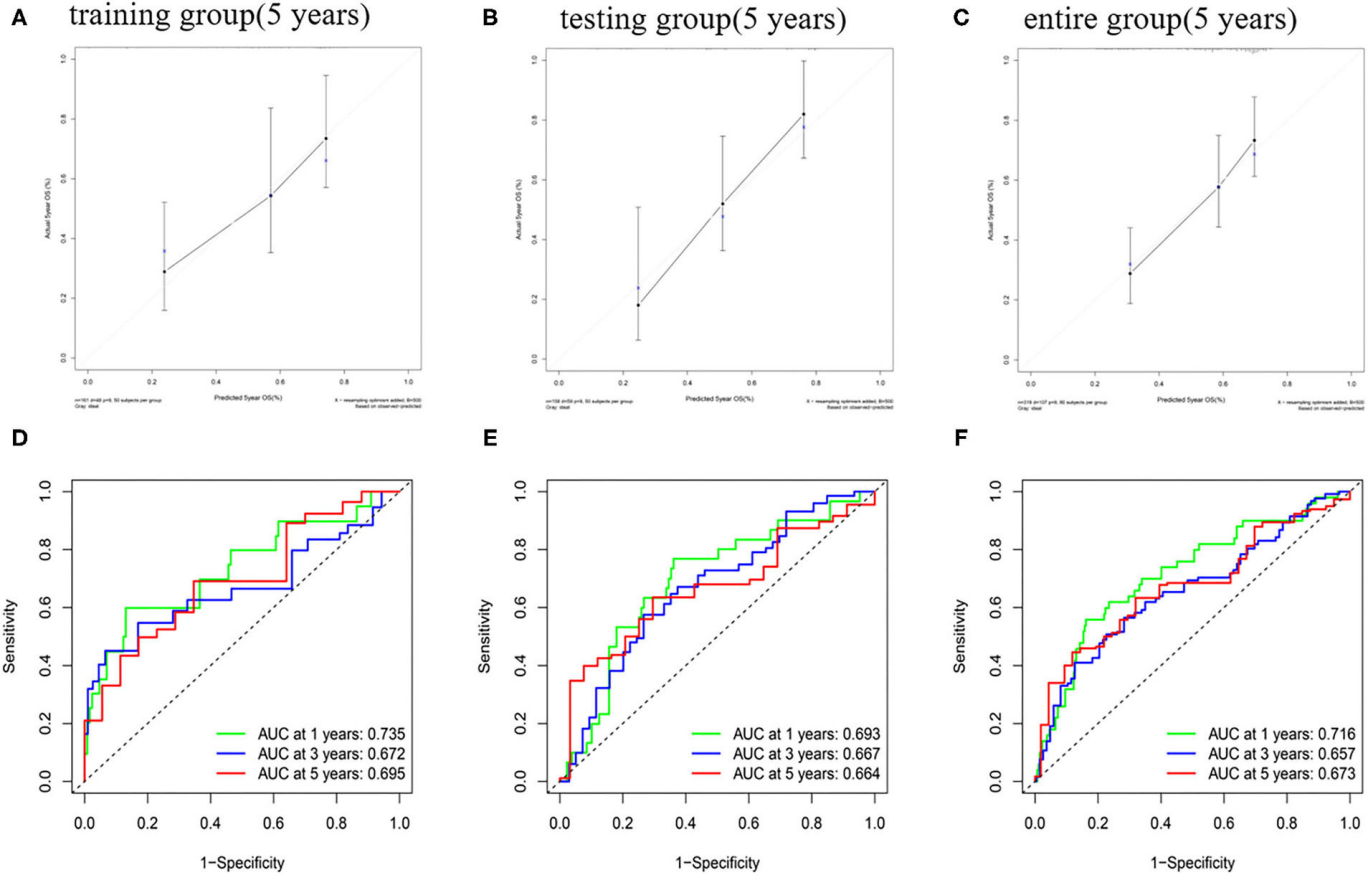
The calibration curve of OS and ROC curve in the training group **(A,D)**, testing group **(B,E)**, and entire group **(C,F)**.

The predictive ability of the nomogram model could be evaluated and quantified by measuring the extent of fit between the C-index forecast by the nomogram in the standard curve and the baseline time. The C-index in the training group was 0.770 (95% *CI*: 0.708–0.832), while that for the testing group and the whole group was 0.680 (95% *CI*: 0.610–0.750) and 0.686 (95% *CI*: 0.635–0.737), respectively. The calibration curves of the nomogram were remarkably consistent between the predicted OS rates and actual observations made at the 5th year in different groups (Figures 8A–C). Simultaneously, the ROC curve analysis showed that AUC was 0.735 after 1 year, 0.672 after 2 years, and 0.695 after 5 years in the training group (Figure 8D). The testing group and the whole group showed similar outcomes (Figures 8E,F).

## Discussion

Hepatocellular carcinoma is a malignant tumor with a highly heterogeneous immune microenvironment, gene expression profile and associated genetic variations, signal transduction pathways, and cancer stem cells (1, 25). The high heterogeneity of liver cancer poses a significant challenge for clinical treatment (6, 7). Tumor heterogeneity may result from genomic instability (26). Genomic instability is a common feature of most cancers and can act as a mutator, enhancing the frequency of mutations that extend the ability of the primary tumor to adapt, escape, and metastasize, ultimately contributing to tumor-specific immune response and resistant phenotypes (27, 28). Thus, genomic instability leads to tumor heterogeneity, which may act as a target for prognosis, prevention, and treatment (29). However, the quantitative analysis of genomic instability is a major problem. An emerging study illustrated that abnormal transcriptional or epigenetic changes may lead to genomic alterations (29, 30). LncRNAs exert a significant effect on the progression of liver cancer, such as regulating proliferation, migration, apoptosis, cell cycle, tumorigenesis, and metastasis (30, 31). An examination of the functional mechanism of lncRNA has shown that lncRNAs are also essential for genomic stability (30, 31).

In this study, the clinical outcomes of the patients with HCC were predicted by exploring the GILncSig. Patients with HCC, whose data are included in the TCGA database, can be distinguished effectively into high-risk and low-risk cohorts by applying the prognostic model risk scores. The Cox analysis showed that the prognostic GILncSig was an independent factor that could effectively predict HCC prognosis better than other clinical factors. A nomogram model was built for the training group. The outcomes of the C-index and time-dependent ROC curves illustrated satisfactory discrimination capacity. The calibration curves showed that the prognosis for the patients with HCC could be predicted by the nomogram with satisfactory performance. Therefore, GILncSig is a promising biomarker for forecasting outcomes in patients with liver cancer.

Specific genes can regulate immune pathways and interactions between immune cells, leading to changes in the microenvironment, allowing tumors to evade immune checkpoints. For instance, a recent study revealed that a specific lncRNA, ALAL-1, associated with genomic instability, mediates evasion of the immune system in the lung cancer cells (32). Here, the relationship between the genomic instability-associated risk model and the main immune system-associated factors was estimated using the ssGSEA analysis. A total of 21 out of 29 patients were significantly altered between patients with low- and high-risk. The terms associated with immune checkpoints, APC co-inhibition and co-stimulation, HLA expression, and Tregs were of particular interest. HLA is a gene cluster encoding the human major histocompatibility complex (MHC) (33). If the peptides provided by HLA proteins are altered due to diseases, they can act as autoantigens that target cellular immune rejection. As shown in Figure 8C, most HLA family genes were expressed significantly in high-risk patients with liver cancer than in low-risk patients. Overexpression of HLA proteins in tumor cells could undermine recognition by the immune system, which accounts for these differences in the survival results among these patient groups.

Administration of immune checkpoint inhibitors (ICIs) is by far the most promising immunotherapeutic strategy (34). Immune checkpoint proteins act as biomarkers that could identify whether patients with liver cancer are suitable for immunotherapy (34). Typically, immune checkpoint molecules can suppress immune responses (35). However, many patients with cancer cannot benefit from immune checkpoint suppression due to abnormal immune checkpoint protein expression (36). The expression of immune checkpoint proteins was significantly higher among patients with high-risk than low-risk patients. The immune response may be suppressed if the immune checkpoint proteins are overexpressed. At the same time, insufficient expression of immune checkpoint inhibitors may result in unconstrained harmful immune responses (37, 38).

More importantly, the increase in the expression of PD-1 in tumor-infiltrating lymphocytes is always associated with poor prognosis among patients with HCC (37, 38). PD-1 is a potential biomarker that aids in determining the suitability of immunochemical checkpoint therapy for patients (39). Recent evidence suggests that PD-(L)1 overexpression and genomic instability in tumors are associated with immune checkpoint inhibitor responses (40–42). In the study, the findings demonstrate the promising potential of immunotherapy for patients with HCC.

The current analysis had some limitations. On the one hand, all the data supplied in this research were derived from the TCGA database; on the other hand, although patients were randomly divided into training and testing queues, the contribution of this internal verification method is limited. Further external validation is critical to identify and extend these outcomes as a potential method for developing clinically valuable prognostic signatures.

## Conclusions

In summary, GILncSig showed satisfactory efficiency for HCC prognosis. Furthermore, the association between the risk model and immune infiltration was explored. The data suggest that this predictive model may provide effective markers for evaluating patients with HCC and immunotherapeutic strategies.

## Data Availability Statement

All data and materials used to support the findings of this study are available from the TCGA (http://cancergenome.nih.gov/).

## Author Contributions

CG, JZha, and ZhiW designed the study. CG, BM, BJ, YG, ZheW, WW, and RW complicated and analyzed data. CG and JZho wrote this manuscript.

## Conflict of Interest

The authors declare that the research was conducted in the absence of any commercial or financial relationships that could be construed as a potential conflict of interest.

## Publisher's Note

All claims expressed in this article are solely those of the authors and do not necessarily represent those of their affiliated organizations, or those of the publisher, the editors and the reviewers. Any product that may be evaluated in this article, or claim that may be made by its manufacturer, is not guaranteed or endorsed by the publisher.

## Abbreviations

HCC: hepatocellular carcinoma
LncRNA: long non-coding RNA
TCGA: The Cancer Genomic Atlas
K-M: Kaplan-Meier
ROC: receiver operating characteristic
FDA: Food and Drug Administration
GU: genomic unstable
GS: genomic stable
GILncSig: genomic instability-related lncRNA signature
FDR: false discovery rate
OS: overall survival
C-index: the concordance index
SNP: single nucleotide polymorphism
AUC: the area under the curve
AFP: alpha-fetoprotein
PD-1: programmed cell death protein-1
PD-L1: programmed cell death protein ligand 1
MHC: major histocompatibility complex
ICI: immune checkpoint inhibitors.
